# Plasma microRNA-320, microRNA-let-7e and microRNA-21 as novel potential biomarkers for the detection of retinoblastoma

**DOI:** 10.3892/br.2014.246

**Published:** 2014-03-07

**Authors:** SHAN-SHAN LIU, YU-SHI WANG, YAN-FENG SUN, LI-XIA MIAO, JUN WANG, YAN-SHAN LI, HONG-YAN LIU, QIU-LING LIU

**Affiliations:** 1 Graduate Division, Xinxiang Medical University, Xinxiang, Henan 453003, P.R. China; 2 Department of Pediatrics, General Hospital of Chinese People’s Armed Police Forces, Beijing 100039, P.R. China; 3 Beijing Institute of Radiation Medicine, Beijing 100850, P.R. China; 4 College of Life Sciences, Jilin University, Changchun, Jilin 130012, P.R. China

**Keywords:** retinoblastoma, microRNA, plasma, biomarker

## Abstract

Retinoblastoma (RB) is a childhood malignancy caused by inactivation of the *RB* gene, with neuron-specific enolase (NSE) levels considered as its diagnostic marker. MicroRNAs (miRNAs) have been proven to play a significant role in multiple physiological and pathological processes and several miRNAs were identified as tumor biomarkers in recent studies. In the present study, 65 plasma samples were collected from RB patients and 65 samples from healthy individuals to serve as controls. The miRNA levels were measured via quantitative reverse transcription-polymerase chain reaction and their association with RB was assessed by statistical data analysis and receiver operating characteristic curves. Plasma miRNA (miR)-320, miR-let-7e and miR-21 levels were downregulated in the patient samples, the areas under the curves (AUCs) were 0.548–0.660, whereas the AUCs of combined classifiers were ≥0.990. The plasma miRNA levels, particularly of miR-320, were found to be of value in RB diagnosis and may be considered as novel diagnostic biomarkers.

## Introduction

Retinoblastoma (RB) is the most common childhood malignancy, with a relative incidence of 1/15,000–20,000 live births annually. The inactivation of the *RB* gene is considered as the initiating event in this disease (1). Delayed diagnosis and treatment contribute to the exacerbation and migration of RB (2). Thus, a timely and accurate diagnosis is required for earlier treatment, which may increase the cure and survival rates.

Imaging techniques are widely used for the diagnosis of RB and images of the tumor tissue may confirm the diagnosis of RB via ophthalmoscopy, ultrasonography, computed tomography and magnetic resonance imaging (2). As a diagnostic marker for small-cell lung cancer and neuroblastoma, neuron-specific enolase (NSE) was found to be significantly elevated in the serum of RB patients and is considered to be a clinical diagnostic indicator (3–6).

MicroRNAs (miRNAs) are a class of mature non-coding single-strand RNAs with a length of 22 nucleotides, which play significant roles in multiple physiological and pathological processes, particularly in tumor development and exacerbation (7,8). An increasing number of studies demonstrate that miRNA expression profiles may be specific to certain types of cancer and tumor-derived miRNAs may be stably detected in the plasma or serum. These findings highlight the potential of circulating miRNAs as biomarkers for the diagnosis of cancer (9–12).

miRNA (miR)-let-7e, a member of the let-7 family, was found to be highly associated with the development and progression of RB. A low level of miR-let-7e contributes to the overexpression of the high-mobility group A1 (HMG A1) and high-mobility group A2 (HMG A2) proteins in RB cells, which are considered as promoters of RB (13). The downregulation of the tumor suppressor miR-let-7e was identified as a biomarker in lung and gastric cancers, uterine leiomyoma and pituitary adenomas (14–17). miR-21 was the first miRNA identified as a diagnostic biomarker, due to its elevated levels in diffuse large B-cell lymphoma (18). miR-320 was suggested to act as a tumor suppressor by inhibiting β-catenin expression via binding to the 3′-untranslated region of β-catenin mRNA in prostate cancer (19). However, it was previously demonstrated that the levels of miR-320 and miR-let-7e were significantly higher in RB compared to those in the normal human retina, according to the results of a miRNA microarray assay (20). Taking into consideration the results of that microarray assay and the fact that miR-21 has been investigated in several types of cancer as a biomarker (21–24), we hypothesized that miR-let-7e, miR-21 and miR-320 may serve as non-invasive circulating biomarkers for the diagnosis of RB. The expression of these 3 plasma miRNAs and the serum NSE levels were measured in RB patients and control subjects matched to the patients by age and gender.

## Materials and methods

### Patients and samples

Blood samples were collected from consenting individuals according to protocols approved by the Institutional Review Board of the General Hospital of the Chinese People’s Armed Police Forces (Beijing, China). Between March, 2012 and June, 2013, a total of 65 patients with RB who had not received any prior treatment and 65 healthy age- and gender-matched controls were enrolled in this study. All the samples were collected once informed consent was obtained from the patients or the legal guardian.

### Sample processing and total RNA extraction

Cell-free plasma was isolated via a two-step protocol (2,500 rpm at room temperature for 10 min and 14,000 × g at 4°C for 10 min) within 2 h after collection to prevent the contamination of cellular nucleic acids. The resulting plasma was transferred to new tubes and stored at −80°C. Total RNA was extracted from 300 μl plasma with the mirVana™ PARIS™ kit (Ambion, Inc., Foster City, CA, USA) according to the manufacturer’s instructions and eluted with 50 μl elution solution pre-heated at 95°C. The RNA quality and concentration was assessed with a K5500 spectrophotometer (Beijing Kaiao Technology Development Co., Ltd., Beijing, China). The concentration of the RNA extracted from plasma was 3.9–18.3 ng/μl.

### Quantitative reverse transcription-polymerase chain reaction (qRT-PCR)

Total RNA was polyadenylated by poly(A) polymerase (New England BioLabs, Inc., Ipswich, MA, USA) and reverse-transcribed to cDNA with the Promega reverse transcription kit (Promega, Madison, WI, USA) according to the manufacturer’s instructions. The reaction mixture for reverse transcription contained 8 μl RNA extract, 2 μl reverse transcription primer (1 μg/μl), 8 μl Improm-II™ 5X reaction buffer, 4.8 μl MgCl_2_ (25 mmol/l), 2 μl dNTPs (10 and 2.5 mmol/l each), 1 μl Recombinant RNasin^®^ Ribonuclease inhibitor (40 U/μl), 2 μl ImProm-II™ Reverse Transcriptase (15 U/μl) and 12.2 μl nuclease-free water to a final volume of 40 μl. The reaction mixtures were incubated at 70°C for 15 min, at 42°C for 60 sec and at 25°C for 5 min and the products were stored at −20°C.

qRT-PCR was performed in a 20-μl reaction containing 10 μl 2X QuantiTect SYBR-Green PCR Master mix (Qiagen, Hilden, Germany), 1 μl gene-specific primers (20 mmol/l), 1 μl cDNA solution and 8 μl nuclease-free water. The reaction mixtures were incubated at 95°C for 15 min, followed by 40 cycles at 95°C for 10 sec, at 60°C for 30 sec and at 72°C for 30 sec, running on a Mx3000P™ thermocycler (Agilent Technologies, Inc., Santa Clara, CA, USA). The primer sequences are presented in Table I.

### Statistical analysis

The qRT-PCR data were analyzed by MxPro software (Agilent Technologies, Inc.) and the normalization was performed with U6 small nuclear RNA. The of miRNA contents were calculated using the formula ΔCt_miRNA_ = Ct_miRNA_ - Ct_U6_. A two-sided χ^2^ test and independent t-tests were used to compare the differences by gender, age, laterality and NSE levels between RB patients and healthy controls. The Mann-Whitney U test was used for the analyses of the expression of different miRNAs. Receiver operating characteristic (ROC) curves were drawn and the areas under the ROC curves (AUCs) were measured to assess the specificity and sensitivity of circulating miRNAs as diagnostic biomarkers for RB. P<0.05 was considered to indicate a statistically significant difference. Statistical analyses were performed with SPSS 17.0 software (SPSS, Inc., Chicago, IL, USA), the ROC curves were generated by MedCalc 12.7.0.0 (http://www.medcale.org; accessed July 10, 2013) and Adobe^®^ Photoshop^®^ CS6 (http://www.adobe.com, accessed February 02, 2013) and the graphs were generated by GraphPad 5.0 software (GraphPad software Inc., La Jolla, CA, USA).

## Results

### Patient characteristics

The clinical characteristics of 65 patients diagnosed with RB were recorded and 65 healthy subjects were recruited as controls. The individual characteristics, including gender, age, laterality and clinical stage are summarized in Table II. The NSE levels were significantly higher in the patient group (27.4±7.0 ng/mL) compared to those in the control group (10.6±3.5 ng/mL). There were no significant differences between the individual characteristics of the patients and those of the control subjects.

### Initiatory screening of plasma miRNAs for the detection of RB

**Figure 1:**
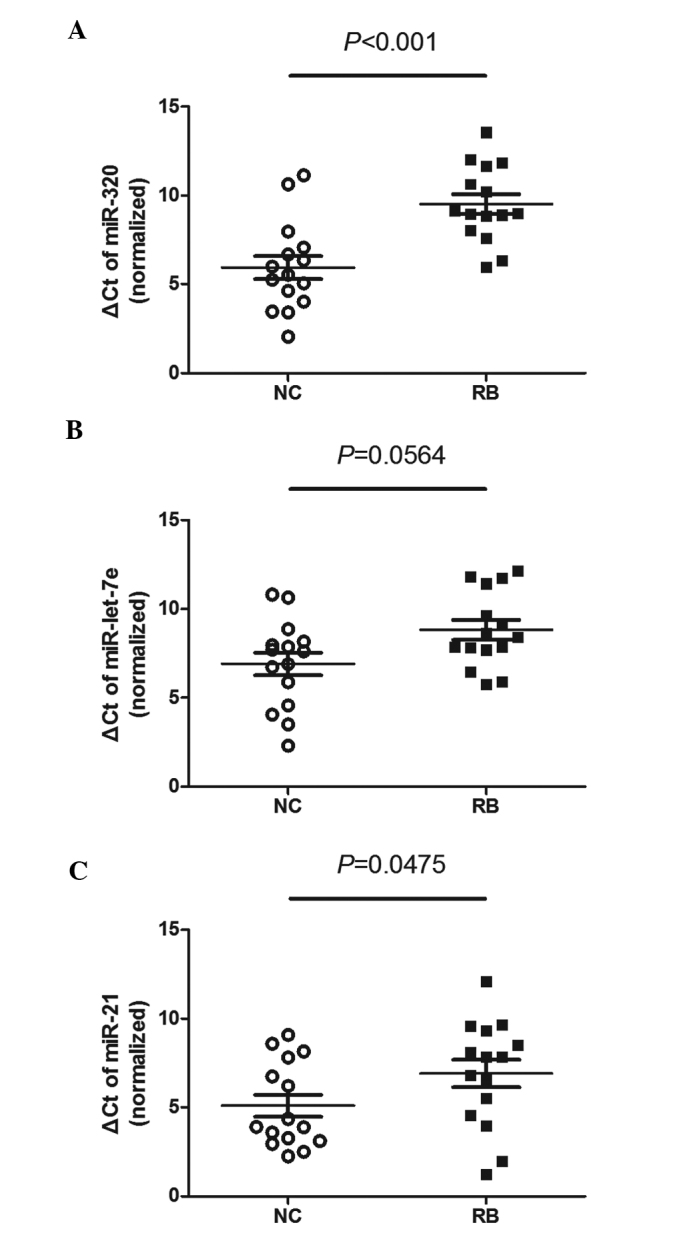
Relative expression levels of plasma miRNAs in initiatory screening. Scatter dot plots of the levels of the 3 plasma miRNAs in retinoblastoma patients (RB, n=15) and normal control subjects (NC, n=15). The scatter dot plots reflect the plasma levels of (A) miR-320, (B) miR-let-7e and (C) miR-21. The lines in the scatter dot plots denote the medians. The miRNA plasma levels were lower in RB patients compared to those in controls (higher relative ΔCt to NC). miRNA, microRNA.

We measured the different miRNA contents in 30 plasma samples (15 patients and 15 healthy controls). The ΔCt of miR-320 in patient plasma was higher compared to that in the control samples. Similar results were found for miR-let-7e and miR-21 (Fig. 1).

### Validation of miR-320, miR-let-7e and miR-21 in a larger sample size

**Figure 2:**
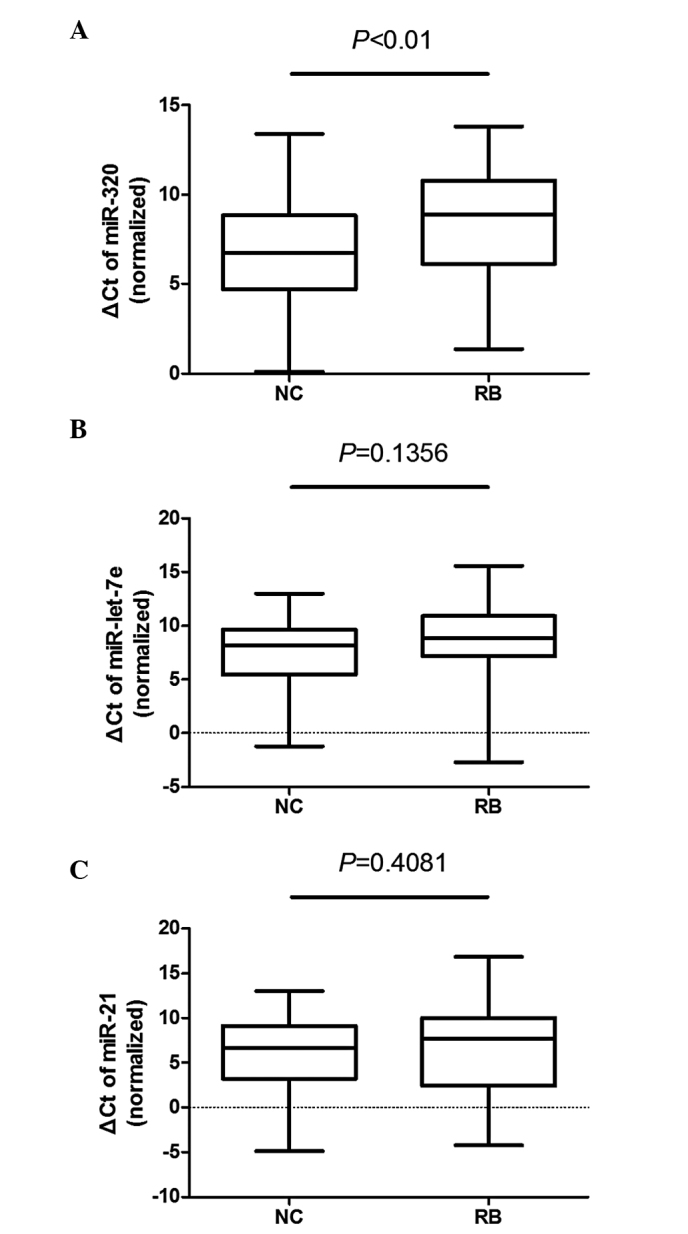
Relative expression levels of plasma miRNAs in initiatory screening. Box plots of the levels of the 3 plasma miRNAs in retinoblastoma patients (RB, n=50) and normal control subjects (NC, n=50). The box plots reflect the plasma levels of (A) miR-320, (B) miR-let-7e and (C) miR-21. The lines in the box plots denote the medians. The miRNA plasma levels were lower in RB patients compared to those in controls (higher relative ΔCt to NC). miRNA, microRNA.

The content of miR-320, miR-let-7e and miR-21 was measured in 100 plasma samples (50 patients and 50 healthy controls). The ΔCt of miR-320 was significantly different between the plasma samples of patients and controls (P<0.01), whereas the differences in miR-let-7e (P=0.1356) and miR-21 (P=0.4081) were not as significant (Fig. 2).

### Diagnostic potential of plasma miRNA and NSE in RB patients

**Figure 3:**
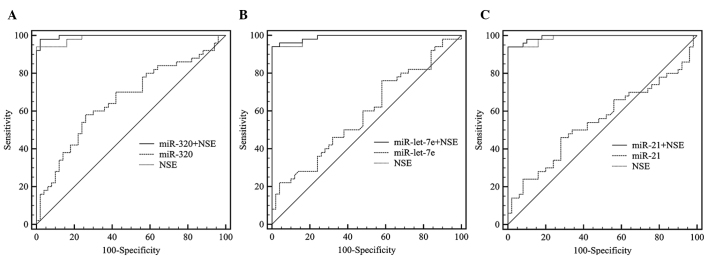
Receiver operating characteristic curves of plasma miRNAs and combined classifiers with NSE. (A) miR-320, (B) miR-let-7e and (C) miR-21 are shown as sparse dotted lines, their combined classifiers with NSE are shown as full lines and the dense dotted lines represent NSE. NSE, neuron-specific enolase. miRNA, microRNA.

ROC curves were used to assess the diagnostic potential of miR-320, miR-let-7e and miR-21 and AUCs indicated its accuracy and reliability. The AUC of NSE reached 0.989 with a cut-off of 15.9. The AUCs of the 3 miRNAs did not reach 70% (Table III) and the ROC curves for the combined classifiers (NSE and miRNAs) were significantly improved compared to those for miRNAs alone. However, the performance of the combined classifier was not significantly improved compared to NSE alone (Fig. 3).

## Discussion

NSE is one of the main diagnostic indicators in the earlier stages of RB. In order to test its accuracy and sensitivity, several clinical characteristics were compared between the patients and healthy controls. Only NSE levels presented a significant difference between cases and controls, whereas individual characteristics, such as age and gender, were similar. Of the 65 patients, >80% were at IIRC clinical stage D-E via NSE level measurement, which indicated that the NSE level was not superior of RB detection at a very early stage (Table II).

miR-320, miR-let-7e and miR-21 were found to be downregulated in the patient group (Fig. 1) and the plasma levels of these 3 miRNAs were also found to be low when the sample size was expanded to 100 subjects (Fig. 2), with the miR-320 level being significantly lower compared to that in normal subjects. The ROC curves for the miRNAs revealed a weaker diagnostic performance for each miRNA alone, although the P-value was of some value for the diagnosis of RB. The combined classifiers also demonstrated the unreliability. However, an elevation of 0.2–0.7% significantly lowers the inaccuracy and possibility of misdiagnosis, with a diagnostic value of 98.9%. The plasma miR-320 exhibited the highest diagnostic value among the 3 investigated miRNAs (P<0.0001: AUC for combined classifier with NSE, 99.6%) and may be considered as a novel plasma biomarker for the diagnosis of RB.

It was reported that a delayed diagnosis of 6 months of RB may increase the mortality by 70% (25); therefore, a biomarker for RB detection at an earlier stage may enable treatment prior to exacerbation, with a lower risk and a higher cure rate. The stability and accuracy of tissue biomarkers are highly associated with the mechanisms underlying tumor development and growth; however, the chances of obtaining a tissue sample when there is no evidence of cancer are limited. Therefore, biomarkers in body fluids are crucial for the early diagnosis of cancer and biomarkers in the serum and plasma are increasingly investigated as diagnostic markers. The NSE level was found to be higher in the serum of RB patients compared to those in control subjects and has become one of the most widely used diagnostic tools for the early diagnosis of RB. The accuracy, sensitivity and reliability of NSE have been extensively investigated based on clinical data (26–28).

Serum and plasma miRNAs are considered as potential biomarkers in several types of cancer; however, their performance is not as satisfactory as that of traditional markers, such as NSE, for RB. In the present study, the plasma miR-320, miR-let-7e and miR-21 levels were found to be lower in RB patients compared to those in healthy control subjects (Fig. 2), whereas their expression in RB tissue was reported to be significantly higher (29). AUC, sensitivity and specificity were not found to be adequate for an accurate prediction on their own. However, combined classifiers with NSE may improve the diagnostic sensitivity and specificity of individual biomarkers to a certain extent, provided that the plasma miRNA levels are of value for the diagnosis of RB. However, further studies are required to assess the reliability and accuracy of miR-320, miR-let-7e and miR-21 as plasma biomarkers of RB.

## Figures and Tables

**Table I:** Primers used for qRT-PCR.

Primers	Sequences (5′-3′)
RT	GCGAGCACAGAATTAATACGACTC ACTATAGG(T)18VN
U6	
Forward	CGCTTCGGCAGCACATATACTA
Reverse	CGCTTCACGAATTTGCGTGTCA
miR-320	AAAAGCTGGGTTGAGAGGGCGA
miR-let-7e	TGAGGTAGGAGGTTGTATAGTT
miR-21	TAGCTTATCAGACTGATGTTGA
3′ universal	GCGAGCACAGAATTAATACGAC

qRT-PCR, reverse transcription-quantitative polymerase chain reaction; miR, microRNA.

**Table II:** Clinical characteristics of the retinoblastoma patients and healthy control subjects.

Characteristics	Cases (n=65)	Controls (n=65)	P-value
Average age, months (mean ± SD)	24.6±16.5	27.92±12.03	0.196^a^
Gender, n (%)			1.000^b^
Male	39 (60.0)	31 (47.7)	
Female	26 (40.0)	34 (52.3)	
Laterality, n (%)			
Unilateral	45 (69.2)	N/A	
Bilateral	20 (30.8)	N/A	
IIRC clinical stage, n			
Group A-C	12	N/A	
Group D-E	53	N/A	
NSE level, ng/mL (mean ± SD)	27.4±7.0	10.6±3.5	<0.0001^a^

a Independent t-test;

b Pearson χ^2^ test.

IIRC, International Intraocular Retinoblastoma Classification; N/A, not applicable; NSE, neuron-specific enolase.

**Table III:** Receiver operating characteristic curve are shown for the 3 miRNAs detected in the plasma samples and their combinations with NSE.

miRNAs, NSE and combinations	AUC	95% CI	Sensitivity (%)	Specificity (%)	P-value
miR-320	0.660	0.558–0.752	58	74	0.0036
miR-let-7e	0.587	0.484–0.684	76	42	0.1280
miR-21	0.548	0.446–0.648	46	72	0.4100
NSE	0.989	0.944–1.000	94	100	<0.0001
miR-320 and NSE	0.996	0.957–1.000	98	98	<0.0001
miR-let-7e and NSE	0.991	0.948–1.000	94	100	<0.0001
miR-21 and NSE	0.993	0.950–1.000	94	100	<0.0001

NSE, neuron-specific enolase; AUC, area under the concentration-time curve; CI confidence interval; miRNA, microRNA.
